# Colonic Stenting Before Endoscopic Ultrasound–Guided Enterocolostomy in Malignant Small Bowel Obstruction

**DOI:** 10.14309/crj.0000000000001671

**Published:** 2025-04-19

**Authors:** Ritik Mahaveer Goyal, Sameer Rao, Anand Shah, Shivani Patel, Imran Qureshi, Ahmed Al-Khazraji, Kaveh Hajifathalian

**Affiliations:** 1 Department of Medicine, Rutgers New Jersey Medical School, Newark, NJ; 2 Division of Gastroenterology and Hepatology, Rutgers New Jersey Medical School, Newark, NJ

**Keywords:** malignant small bowel obstruction, colonic stenting, enterocolostomy, palliative care

## Abstract

Malignant Small Bowel Obstruction (MSBO) is a debilitating complication of intra-abdominal malignancies, characterized by symptoms such as intractable nausea, vomiting, and abdominal pain. Surgical intervention may become necessary for symptom management if conservative measures fail, although it carries a significant risk of postoperative morbidity. Endoscopic ultrasound–guided enterocolostomy is an emerging alternative for palliative management of MSBO when other surgical or endoscopic interventions are not feasible or have failed. We present a case of a 50-year-old woman with MSBO secondary to metastatic squamous cell carcinoma of cervix who required the placement of a luminal colonic stent in the sigmoid colon before undergoing endoscopic ultrasound–guided enterocolostomy.

## INTRODUCTION

Malignant Small Bowel Obstruction (MSBO) can be broadly defined as a mechanical or functional obstruction of the intestinal tract, accompanied by clinical evidence of obstruction, in the setting of a primary or metastatic intra-abdominal malignancy.^1^ It is a common complication of gastrointestinal (GI) and gynecological cancers, affecting 10%–28% of GI malignancies and occurring more frequently in gynecological cancers.^2,3^ Patients typically present with symptoms such as abdominal pain, abdominal distension, nausea, vomiting, constipation, and loss of appetite. Initial treatment focuses on conservative management, but surgical or endoscopic intervention may become necessary if these measures fail.^4^ We present a case of MSBO who required placement of a luminal colonic stent in the sigmoid colon before an endoscopic ultrasound–guided entero-colostomy (EUS-EC) to relieve the obstruction.

## CASE REPORT

A 50-year-old Hispanic woman with a medical history of moderate to poorly differentiated stage 4 squamous cell carcinoma of the cervix, treated with 6 cycles of chemoradiotherapy and 4 doses of brachytherapy, complicated by peritoneal carcinomatosis, presented to the emergency department with abdominal pain, nausea, vomiting, and no bowel movement for 5 days. A computed tomography (CT) scan of the abdomen revealed multiple dilated fluid-filled small bowel loops with a transition point in the right lower quadrant, consistent with a small bowel obstruction. She underwent diagnostic laparoscopy, lysis of adhesions, and biopsy of peritoneal implants and mesenteric nodules. Pathology confirmed metastatic keratinizing squamous cell carcinoma.

**Figure 1.**
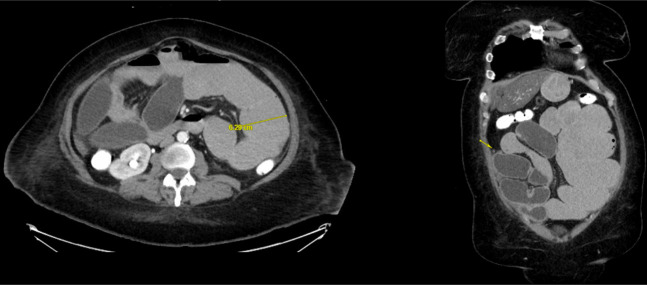
Computed tomography of the abdomen and pelvis showing multiple dilated small bowel loops measuring up to 6.3 cm and a transition point in the right lower quadrant, indicating a possible closed-loop obstruction in the ileum.

Over the following months, she presented to the emergency department several times with similar complaints of nausea, vomiting, and constipation. Conservative management with bowel rest, nasogastric tube decompression, and intravenous (IV) fluids provided only partial symptom relief. A repeat CT scan of the abdomen with IV contrast showed findings similar to those from 2 months prior, with multiple dilated small bowel loops measuring up to 6.3 cm and a transition point in the right lower quadrant, indicating a possible closed-loop obstruction in the ileum (Figure 1).

**Figure 2.**
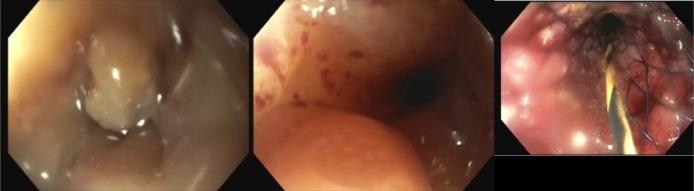
Sigmoid stenosis on colonoscopy and wall-flex stent placement.

**Figure 3.**
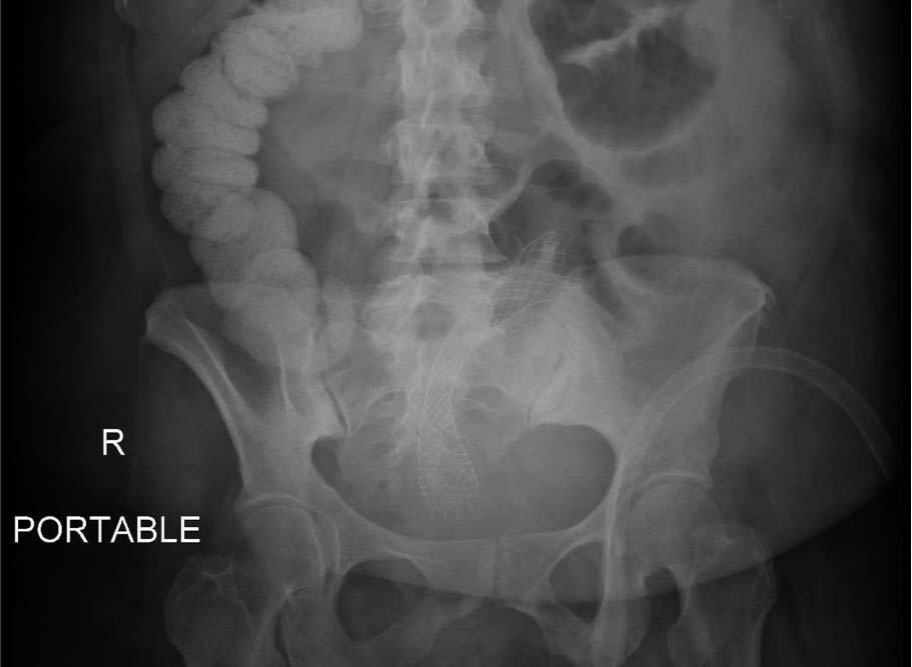
X-ray abdomen showing luminal sigmoid stent.

Given the patient's advanced metastatic disease and history of radiotherapy, surgical intervention was deemed too risky due to concerns about poor healing and recovery. As a palliative measure, an EUS-EC was planned to connect the jejunum to the descending colon, as the dilated bowel loops were adjacent to this part of the colon. During colonoscopy, a severe, malignant-appearing intrinsic stenosis of unknown length was identified in the sigmoid colon. Fluoroscopy was used to assess its extent, and a 25 × 60-mm WallFlex stent with anchor lock delivery was placed under fluoroscopic guidance to traverse the stenosis (Figure 2). However, this rendered the EC procedure unfeasible at the time. Postprocedure, the patient experienced loose stools and nausea but no abdominal pain or vomiting. Daily abdominal X-rays were performed to monitor stent dilation; however, the stent continued to show narrowing, measuring up to 1.2 cm (Figure 3).

**Figure 4.**
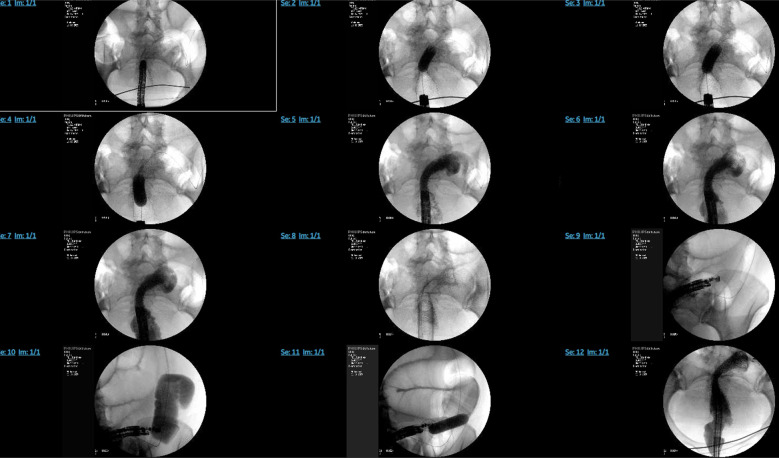
Dilation of the wall-flex stent in the sigmoid colon with a 15–16.5–18 mm colonic balloon dilator under fluoroscopic guidance.

**Figure 5.**
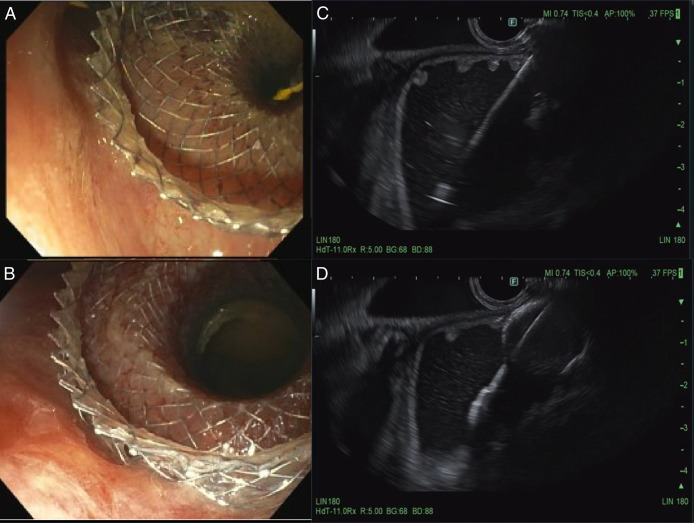
(A, B) Endoscopic images of an Axios stent deployed from the distal descending colon toward likely mid-jejunum, (C) EUS image before stent deployment (endoscopy within the descending colon lumen, which is seen above the jejunum lumen), and (D) EUS image after stent deployment. EUS, endoscopic ultrasound.

One week later, an attempt was made to perform the EC. Due to difficulty passing a double-channel colonoscope through the wall-flex stent, a forward viewing single channel Olympus endoscope (serial number–2854657) was used instead. A guidewire was passed through, followed by a through-the-scope dilator. The wall-flex stent in the sigmoid colon was dilated using a 15–16.5–18 mm colonic balloon dilator under fluoroscopic guidance (Figure 4). The endoscope was then removed and reinserted with a capped overtube, advancing to the sigmoid colon to facilitate repeated scope passages. An EUS scope was then passed through the overtube and colonic stent into the distal descending colon, which appeared normal, along with adjacent dilated small bowel loops. An Axios stent was deployed between the distal descending colon and likely mid-jejunum under EUS and fluoroscopy guidance (Figure 5). Three hemostatic clips were placed to secure the stent, and contrast dye was used to confirm stent placement and rule out perforation.

Poststenting, the patient reported mild left lower quadrant pain but was able to pass flatus and had a small bowel movement with blood and mucus. She was started on a clear liquid diet on the third day, which she tolerated well, and subsequently advanced to a regular diet, also well tolerated despite watery diarrhea. She was prescribed loperamide, fiber supplementation, and Metamucil to manage the diarrhea. The patient was discharged with dietary instructions.

**Figure 6.**
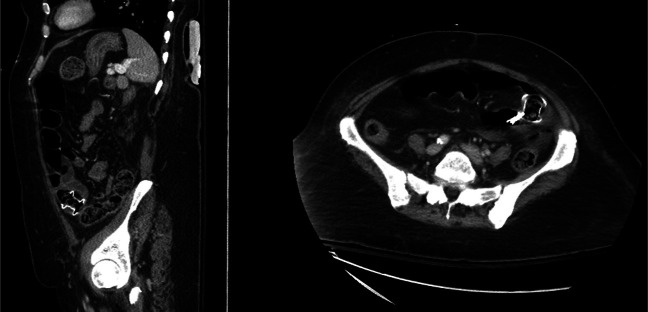
Computed tomography of the abdomen showing lumen apposing stent placement.

Unfortunately, the patient died 4 months later due to neutropenia and sepsis. During the intervening period, she did not experience significant GI issues. A follow-up CT abdomen confirmed that the lumen-apposing stent remained well positioned, and her GI symptoms were well controlled (Figure 6).

## DISCUSSION

The management of MSBO is complex, requiring consideration of multiple factors such as cancer stage, prognosis, quality of life, and the wishes of the patient and their family. The primary objective is to treat these patients holistically, prioritizing the patient's quality of life. Initial management typically includes conservative measures like bowel rest, nasogastric decompression, IV fluids, avoiding medications that alter GI motility, and correcting biochemical abnormalities.^4^ If symptoms are not controlled with these approaches, surgical interventions such as intestinal resection, bypass, creation of an ostomy, adhesiolysis, or the percutaneous placement of a gastrostomy or long jejunal tube may be considered. A systematic review has shown that surgical interventions can alleviate obstructive symptoms in 32%–100% of patients, although these procedures carry a significant complication rate, ranging from 7% to 44%.^5^ In addition, a substantial proportion of patients, particularly those with extensive disease and poor overall health, may not be suitable candidates for surgery.

Recent advancements have explored endoscopic approaches, such as EUS-guided anastomosis with lumen-apposing metal stent, as potential options for managing MSBO. Retrospective studies, although very limited in sample size, have reported immediate clinical success rates ranging from 70% to 92.3%, with relatively low adverse event rates, suggesting its potential as an alternative therapeutic option.^6–8^

In our case, we used luminal colonic stenting to address severe sigmoid stenosis, followed by an EUS-guided jejunum-to-colon anastomosis with a lumen-apposing metal stent to relieve MSBO in the ileum. This approach highlights the importance of individualized therapy in managing complex conditions like MSBO, demonstrating how enterocolostomy can be effectively paired with luminal colonic stenting to achieve symptomatic relief in such challenging scenarios. However, further prospective studies with larger sample sizes and extended follow-up periods are essential to more comprehensively assess the short-term and long-term impacts of this approach on patient morbidity and mortality.

## DISCLOSURES

Author contributions: RM Goyal: data curation, writing–original draft, literature review. S, Rao: writing–original draft, literature review. A. Shah: data curation, writing–review and editing. S. Patel and I. Qureshi: literature review, writing–review and editing. A. Al-Khazraji: supervision, writing–review and editing. K. Hajifathalian: project administration, supervision, writing–review and editing. K. Hajifathalian is the article guarantor.

Financial disclosure: None to report.

Previous presentation: Abstract from the case report was presented at American College of Gastroenterology (ACG) Annual Scientific Meeting; October 28–30, 2024; Philadelphia, Pennsylvania.

Informed consent was obtained for this case report.
